# Effects of Compressive and Tensile Strain on Macrophages during Simulated Orthodontic Tooth Movement

**DOI:** 10.1155/2020/2814015

**Published:** 2020-04-28

**Authors:** Agnes Schröder, Paul Käppler, Ute Nazet, Jonathan Jantsch, Peter Proff, Fabian Cieplik, James Deschner, Christian Kirschneck

**Affiliations:** ^1^Department of Orthodontics, University Hospital Regensburg, 93053 Regensburg, Germany; ^2^Institute of Clinical Microbiology and Hygiene, University Hospital Regensburg, 93053 Regensburg, Germany; ^3^Department of Operative Dentistry and Periodontology, University Hospital Regensburg, 93053 Regensburg, Germany; ^4^Department of Periodontology and Operative Dentistry, University of Mainz, 55131 Mainz, Germany

## Abstract

During orthodontic tooth movement (OTM) to therapeutically correct the position of misaligned teeth, thus improving oral health and quality of life, fibroblasts, macrophages, and other immune cells within the periodontal ligament (PDL), which connects a tooth to its surrounding bone, are exposed to compressive and tensile strain. While it is known that PDL fibroblasts are critically involved in the biological regulation of OTM by a mechanotransductively triggered release of cytokines, it is unclear whether macrophages also react to pressure and tension in a similar manner thus impacting on or mediating OTM. RAW264.7 macrophages were seeded onto conventional 6-well cell culture plates for pressure or on Bioflex plates for tension assays and preincubated for 24 h. For *in vitro* simulation of physiological orthodontic compressive or tensile strain for 2 h, 4 h, 24 h, and 48 h, glass discs (2 g/cm^2^) were placed or adherent macrophages isotropically stretched for 16%, respectively. We determined cell number, cytotoxicity, and gene/protein expression of *Vegf-a*/VEGF-A (macrophage-mediated angiogenesis), *Mmp-8/9* (extracellular matrix reorganization), and *Cox-2*/PG-E2, *Il-6*/IL-6, and *Tnf-α*/TNF-*α* (proinflammatory mediators) by RT-qPCR and ELISA. Compressive but not tensile strain resulted in a significant reduction in cell number after only 2 h. *Mmp-8* and *Mmp-9* expression was significantly enhanced within 24 h of compressive and in part tensile strain. Significantly increased *Vegf-a*/VEGF-A expression was detected within 4 h of pressure, but not during application of tensile strain. Expression of proinflammatory mediators *Cox-2*/PG-E2, *Il-6*/IL-6, and *Tnf-α*/TNF-*α* was significantly increased as early as 2-4 h after application of compressive or tensile strain. Our results indicate that macrophages respond early on to compressive and tensile strain occurring during OTM with an enhanced gene expression of proinflammatory cytokines, which could affect PDL fibroblasts, osteoblasts, and immune cells triggering or enhancing the biological mechanisms and osteoclastogenesis underlying OTM.

## 1. Introduction

The orthodontic correction of malpositioned teeth is of great medical significance, as it was demonstrated that malocclusions of teeth might be associated with an increased prevalence of dental caries [1, 2], gingivitis, periodontitis, and gingival recessions [3, 4]. Particularly, untreated dental caries in deciduous teeth has an extremely high prevalence, currently affecting 532 million children all over the world [5], and thus is referred to as the “most important global health burden” by the WHO [6]. Therefore, orthodontics has an important preventive function for the development and progression of these diseases. To achieve orthodontic tooth movement (OTM) to correct the position of misaligned teeth, a mechanical force is applied to the teeth by means of removable or fixed orthodontic appliances in the direction of movement. This applied force leads to the formation of tensile and pressure zones in the periodontal ligament (PDL), a fibrous and cellular connective tissue anchoring the tooth in the alveolar socket of the jawbone. Despite its fibrous nature, the PDL is a highly cellular structure essential for the long-term health of the masticatory apparatus. After applying an orthodontic force to a tooth, there is an initial tipping within the bony socket and cells in the PDL are subjected to compressive or tensile mechanical strain [7, 8]. The main cell population found in the PDL consists of fibroblasts, but also osteoblasts and immune cells like macrophages, leukocytes, and T cells [9] are also present in the PDL. PDL fibroblasts (PDLF) are known to be responsible for the regulation of tissue homeostasis and the formation of collagenous structural proteins [7, 8]. Furthermore, they perform regulatory functions in innate immune defence [7] and play a major mediating role during OTM. Therefore, they have been intensively investigated in basic orthodontic research [10–14], especially with regard to their responses to compressive or tensile orthodontic forces. Compressive force application increased the synthesis of proinflammatory enzymes, cytokines, and chemokines in PDLF [11, 14, 15]. In particular, prostaglandins were expressed due to an increased activity of cyclooxygenase-2 (COX-2). This promotes an increased expression of soluble and membrane-linked RANK-L (receptor activator of nuclear kappa b ligand) [11] as well as of proinflammatory cytokines like interleukin-1 (IL-1), IL-6, tumor necrosis factor-*α* (TNF-*α*), and chemokines such as IL-8 [14, 16]. These in turn further enhance the expression of RANK-L and increased the release of matrix metalloproteases [17], which promote the degradation of the extracellular matrix [7] thus inducing the transformation of the PDL for OTM. The enhanced release of RANK-L by PDLF [18] increases the ratio of RANK-L to osteoprotegerin (OPG), which acts as a soluble decoy receptor of RANK-L. This promotes osteoclastogenesis and bone resorption at the pressure zones of the PDL [7], since the binding of RANK-L to the RANK receptor on the surface of osteoclast precursor cells is essential for their differentiation into mature osteoclasts [19].

The mediating role of PDL fibroblasts during OTM is well studied; however, the function and role of macrophages receiving compressive or tensile strain in the context of OTM are not yet investigated. In this study, we therefore focused on the expression profile of macrophages exposed to compressive or tensile mechanical strain occurring during OTM for different timeframes, in order to gain a better understanding of the role of macrophages in the transformation processes of the PDL during OTM.

## 2. Materials and Methods

### 2.1. *In Vitro* Cell Culture Experiments

**Figure 1:**
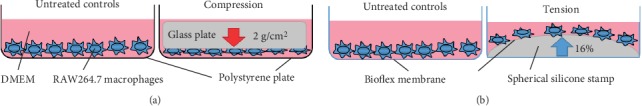
In vitro setup for the application of static compressive (a) and tensile isotropic strain (b). DMEM = Dulbecco‘s modified Eagle medium.

RAW264.7 macrophages were purchased from CLS (400319, Cell Lines Service GmbH, Eppelheim, Germany) and cultured in DMEM high glucose (D5796, Sigma-Aldrich, Munich, Germany) supplemented with 10% FBS (P30-3306, PAN-Biotech, Aidenbach, Germany), 1% L-glutamine (SH30034.01, GE Healthcare Europe, Munich, Germany), and 1% antibiotics/antimycotics (A5955, Sigma-Aldrich). Either 1 × 10^6^ (for 2 h and 4 h incubation), 500,000 (for 24 h incubation), or 250,000 (for 48 h incubation) RAW264.7 macrophages were seeded for pressure experiments onto standard 6-well polystyrene plates (353046, BD, Heidelberg, Germany) or for tensile loading experiments on 6-well Bioflex plates (BF-3001, Dunn Labortechnik, Asbach, Germany) and preincubated for 24 h without any mechanical strain to allow adherence. To simulate orthodontic compressive forces in PDL compression areas, a pressure of 2 g/cm^2^ was applied to the RAW264.7 macrophages under cell culture conditions at 70% confluency for 2 h, 4 h, 24 h, and 48 h, respectively, by means of a glass disc according to an established and published method for simulated orthodontic compressive force application (Figure 1(a)) [10, 12–14]. In parallel, on the same respective 6-well plates, RAW264.7 macrophages were cultivated for 2 h, 4 h, 24 h, and 48 h without compressive forces (control). 16% static isotropic tensile strain was applied to RAW264.7 macrophages under cell culture conditions at 70% confluency by means of spherical silicone stamps inserted from the well bottom into the flexible Bioflex membrane for 2 h, 4 h, 24 h, and 48 h according to an established and published method to simulate tensile orthodontic strain in *in vitro* experiments (Figure 1(b)) [20, 21]. In parallel, RAW264.7 macrophages were again cultured on the same Bioflex 6-well plates without stretching for 2 h, 4 h, 24 h, and 48 h.

### 2.2. Assessment of Cell Number

After the appropriate incubation time, we harvested RAW264.7 macrophages with a cell scraper in 1 ml PBS and quantified the cell number with a Beckman Coulter Counter Z2™ (Beckman Coulter GmbH, Krefeld, Germany) according to the manufacturer's instructions.

### 2.3. Measurement of Cytotoxicity via LDH Assay

We performed lactate dehydrogenase (LDH) assays (04744926001, Roche, Mannheim, Germany) to assess cytotoxicity. For that, we used cell supernatants according to the manufacturer's instructions. Briefly, we mixed 50 *μ*l of the supernatant with 50 *μ*l freshly prepared LDH solution (22 *μ*l catalyst mixed with 1 ml dye) and incubated for 30 min at room temperature in the dark. After addition of 25 *μ*l stop solution, we measured the absorbance at 490 nm (LDH activity) with an ELISA reader (Multiskan GO Microplate Spectrophotometer, Thermo Fisher Scientific) subtracting background absorbance at 690 nm.

### 2.4. Isolation and Purity Assessment of Total RNA

RNA isolation was performed as described before [13]. Briefly, we extracted total RNA from RAW264.7 macrophages by applying 500 *μ*l peqGOLD TriFast™ (PEQLAB, Erlangen, Germany) per well and further processing according to the manufacturer's instructions. We resuspended the resulting pellet in 20 *μ*l nuclease-free water (T143, Carl Roth, Karlsruhe, Germany) and immediately cooled it on ice. We determined optical density photometrically at 280 nm and 260 nm (NanoPhotometer N60, Implen, Munich, Germany) to assess purity and quantity of the eluted total RNA.

### 2.5. Reverse Transcription (cDNA Synthesis)

We used a standardized amount of 100 ng RNA per sample for cDNA synthesis, as well as 0.5 *μ*l random hexamer primer (0.1 nmol, Life Technologies), 0.5 *μ*l oligo-dT18 primer (0.1 nmol, Life Technologies), 2 *μ*l 5x M-MLV buffer (Promega), 0.5 *μ*l dNTP mix (40 nmol, dNTP, Carl-Roth), 0.5 *μ*l RNase inhibitor (40 U, Life Technologies), and 0.5 *μ*l reverse transcriptase (200 U, Promega), and added nuclease-free H_2_O (Carl-Roth) to a final volume of 10 *μ*l. The samples were incubated at 37°C for 60 min, and the reverse transcriptase was heat-inactivated at 95°C for 2 min. To minimize experimental variations, synthesis of cDNA was performed concurrently for all samples. We stored cDNA at −20°C until use.

### 2.6. Quantitative Real-Time Polymerase Chain Reaction (RT-qPCR)

RT-qPCR amplification was performed according to MIQE quality control guidelines as described before [13, 22] with a Mastercycler® ep realplex-S thermocycler (Eppendorf AG, Hamburg, Germany). For RT-qPCR reaction, we used SYBR® Green JumpStart™ Taq ReadyMix™ (7.5 *μ*l, Sigma-Aldrich®, S4438), the respective primer pairs (7.5 pmol, 0.75 *μ*l-3.75 pmol/primer), and 15 *μ*l nuclease-free H_2_O (T143, Carl-Roth GmbH) and added the respective cDNA solution (1.5 *μ*l, dilution 1 : 5). We performed amplification of the cDNA in duplex in 45 cycles (initial heat activation 95°C/5 min, per cycle 95°C/10 s denaturation, 60°C/8 s annealing, and 72°C/8 s extension). At the end of each extension step, SYBR® Green I fluorescence was detected at 521 nm. Primers were synthesized and purified by Eurofins MWG Operon LLC (Huntsville, AL, USA; High Purity Salt Free Purification HPSF®) and were not modified (Table 1). They were constructed using NCBI Primer-BLAST according to MIQE guidelines as described before [13, 20, 22]. For determination of relative gene expression, two reference genes in combination (*EEF1A1* and *SDHA*) were used, which have been shown to be stably expressed in RAW264.7 macrophages under the conditions investigated (data not shown). Relative gene expression was calculated as 2^-*Δ*Cq^ with ΔCq = Cq (target gene) − Cq (*EEF*1*A*1/*SDHA*) [23, 24]. For each primer pair and RT-qPCR run, a no-template control without cDNA was performed.

### 2.7. Enzyme-Linked Immunosorbent Assays (ELISAs)

Cell culture supernatants were stored at -80°C until use and thawed on ice. ELISAs were performed with cell culture supernatants only after 4 h of mechanical strain according to the manufacturer's instructions (Vascular Endothelial Growth Factor-A: MBS043195, Tumor Necrosis Factor-*α*: MBS335449, MyBioSource; Prostaglandin E2: MBS266212, MyBioSource; Interleukin-6: MBS335514, MyBioSource).

### 2.8. Statistical Methods

Prior to statistical analysis, all absolute data values were divided by the respective arithmetic mean of the strain-untreated controls to obtain normalized data values relative to these controls, set to 1. Using GraphPad Prism 8.0 (normalized) relative gene expression during application of compressive/tensile forces across application times including controls was then statistically evaluated by independent nonparametric two-sided Kruskal-Wallis *H* tests. For statistical comparison of compression/tension to control groups at each incubation time (2, 4, 24, and 48 h), Dunn's multiple comparison post hoc tests were used. Data of protein secretion were analysed by independent nonparametric two-sided Mann-Whitney *U* tests. The significance level was set at *p* = 0.05.

## 3. Results

### 3.1. Impact of Pressure and Tension Treatment on Cell Number and Cytotoxicity

**Figure 2:**
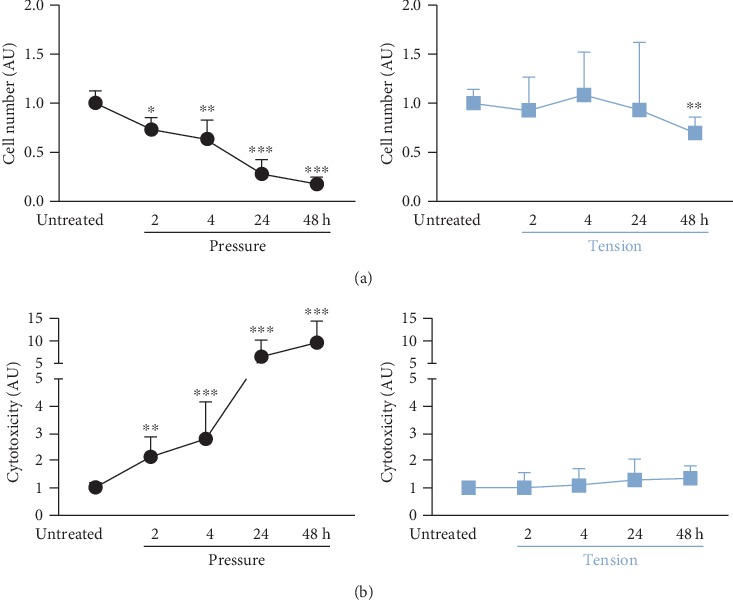
Impact of static pressure application (pressure) or tensile strain (tension) on cell number (a) and cytotoxicity (LDH assay (b)) after 2, 4, 24, or 48 h of treatment. AU: arbitrary units; error bars: standard deviation; untreated: *n* = 35, 2 h–48 h: each *n* = 9; ^∗^*p* ≤ 0.05, ^∗∗^*p* ≤ 0.01, and ^∗∗∗^*p* ≤ 0.001 compared to untreated control. Statistics: Kruskal-Wallis *H* test followed by Dunn's multiple comparison post hoc tests.

Compressive strain had a significant impact on cell survival (*H* = 56.8, *p* < 0.001) and cytotoxicity (*H* = 58.2, *p* < 0.001) of RAW264.7 macrophages. Compressive strain decreased cell number already after 2 h of application time significantly (*p* = 0.012) with a constant reducing effect up to 48 h (*p* < 0.001; Figure 2(a)). This was in line with the results of the cytotoxicity assay. We observed a significantly enhanced release of lactate dehydrogenase (LDH) in macrophages already after 2 h of pressure application (*p* = 0.003) with a maximum effect after 48 h (*p* < 0.001, Figure 2(b)). Despite isotropic tensile strain also having a significant effect on cell number (*H* = 14.6, *p* = 0.006), it caused only a slight, but significant reduction not until 48 h (*p* = 0.004, Figure 2(a)). In line with that, we detected no significant changes in cytotoxicity during the application of tensile strain (*H* = 5.156, *p* = 0.272; Figure 2(b)).

### 3.2. Impact of Pressure and Tension Treatment on Macrophage-Induced Remodelling of the Extracellular Matrix

**Figure 3:**
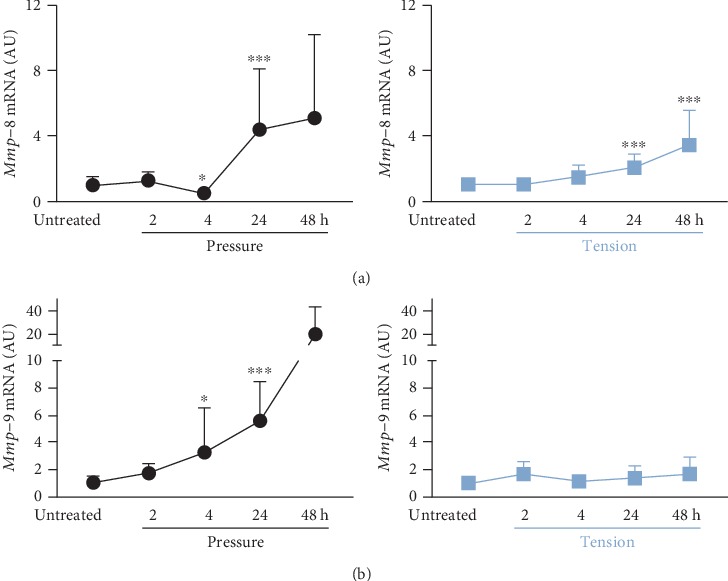
Impact of static pressure application (pressure) or tensile strain (tension) on *Mmp-8* (a) and *Mmp-9* (b) gene expression after 2, 4, 24, or 48 h of treatment. AU: arbitrary units; error bars: standard deviation; untreated: *n* = 35, 2 h–48 h: each *n* = 9; ^∗^*p* ≤ 0.05, ^∗∗^*p* ≤ 0.01, and ^∗∗∗^*p* ≤ 0.001 compared to untreated control. Statistics: Kruskal-Wallis *H* test followed by Dunn's multiple comparison post hoc tests.

Matrix metalloproteinases (MMPs) are responsible for extracellular matrix reorganization. Macrophages reacted to compressive force treatment with enhanced gene expression of *Mmp-8* after 24 h and *Mmp-9* already after 4 h (*Mmp-8*: *p* = 0.001; *Mmp-9*: *p* = 0.037) of application time (*Mmp-8*: *H* = 29.61, *p* < 0.001; *Mmp-9*: *H* = 33.37, *p* < 0.001; Figure 3). In contrast, tensile strain only affected *Mmp-8* (*H* = 34.44, *p* < 0.001) but not *Mmp-9* gene expression in macrophages. We observed a significant increase in *Mmp-8* gene expression after 24 h (*p* < 0.001) and 48 h (*p* < 0.001) of tensile strain (Figure 3(a)). Throughout the studied period of 48 h, we observed no changes in *Mmp-9* gene expression in macrophages upon stretching (*H* = 3.77, *p* = 0.438; Figure 3(b)).

### 3.3. Impact of Pressure and Tension Treatment on Macrophage-Induced Angiogenesis

**Figure 4:**
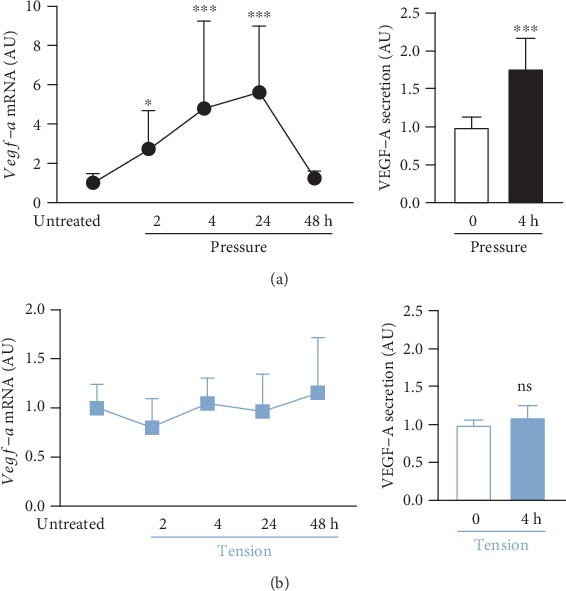
Impact of static pressure application (pressure (a)) or tensile strain (tension (b)) on *Vegf-a*/VEGF-a gene and protein expression after 2, 4, 24, or 48 h of treatment. AU: arbitrary units; error bars: standard deviation; mRNA: untreated: *n* = 35, 2 h–48 h: each *n* = 9; secretion: *n* = 6; ^ns^not significant; ^∗^*p* ≤ 0.05, ^∗∗^*p* ≤ 0.01, and ^∗∗∗^*p* ≤ 0.001 compared to untreated control. Statistics: mRNA: Kruskal-Wallis *H* test followed by Dunn's multiple comparison post hoc tests; secretion: Mann-Whitney *U* test.

Next, we investigated the impact of compressive and tensile strain on macrophage-induced angiogenesis. Vascular endothelial growth factor-a (VEGF-A) is responsible for blood vessel neoformation and adjustment to hypoxic conditions occurring during tooth movement in pressure zones. Compressive force application enhanced *Vegf-a* gene expression significantly after 2 h (*p* = 0.015), 4 h (*p* < 0.001), and 24 h (*p* < 0.001) of pressure treatment (*H* = 37.84, *p* < 0.001; Figure 4(a)). After 48 h of ongoing compression, there was no amplified *Vegf-a* gene expression detectable (*p* = 0.991, Figure 4(a)). Accordingly, we detected a significantly enhanced secretion of VEGF-A after 4 h of compressive force treatment (*p* < 0.001; Figure 4(a)). In contrast to compressive force, we observed no effects of tensile strain on *Vegf-a* gene expression within 48 h in macrophages (*H* = 4.86, *p* = 0.302) or VEGF-A secretion (*p* = 0.240; Figure 4(b)).

### 3.4. Impact of Pressure and Tension Treatment on the Expression of Proinflammatory Genes

**Figure 5:**
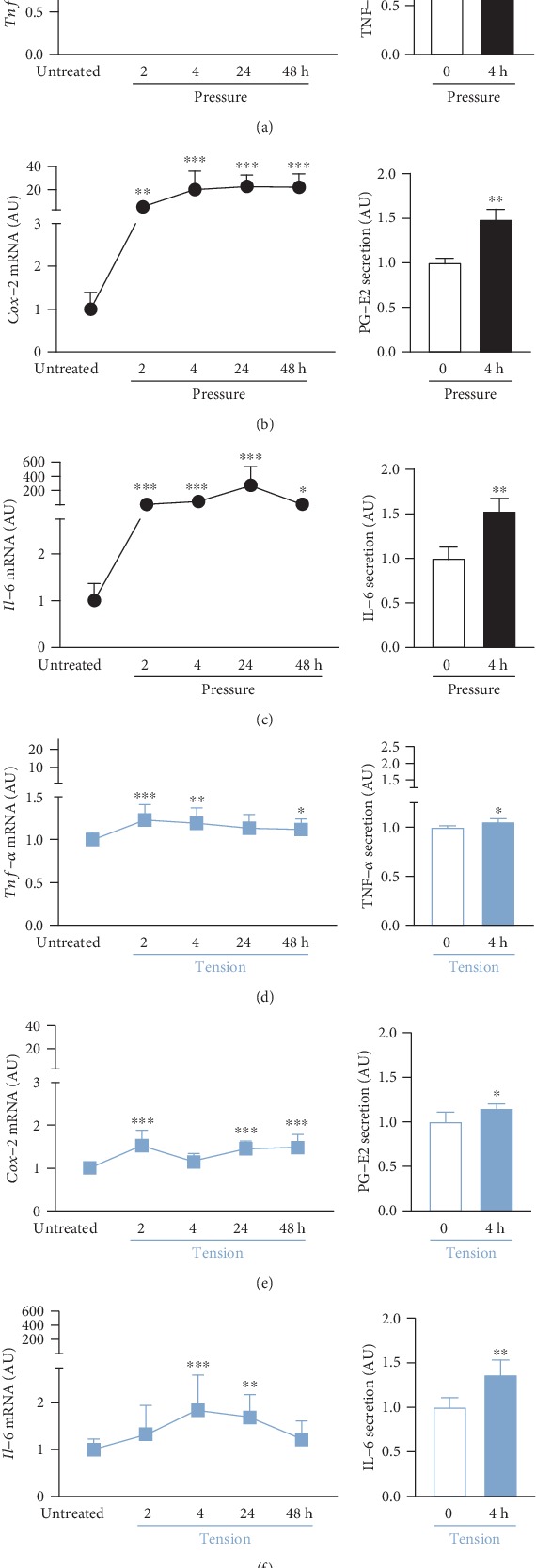
Impact of static pressure application (pressure (a–c)) or tensile strain (tension (d–f)) on *Tnf-α/*TNF-*α* (a, d), *Cox-2/*PG-E2 (b, e), and *Il-6/*IL-6 (c, f) gene and protein expression after 2, 4, 24, or 48 h of treatment. AU: arbitrary units; error bars: standard deviation; mRNA: untreated: *n* = 35, 2 h–48 h: each *n* = 9; secretion: *n* = 6; ^∗^*p* ≤ 0.05, ^∗∗^*p* ≤ 0.01, and ^∗∗∗^*p* ≤ 0.001 compared to untreated control. Statistics: mRNA: Kruskal-Wallis *H* test followed by Dunn's multiple comparison post hoc tests; secretion: Mann-Whitney *U* test.

Compressive force treatment led to an upregulation of *tumor necrosis factor-α* (*Tnf-α*) gene expression already after 2 h (*p* < 0.001) of treatment and a maximum induction after 4 h (*p* < 0.001) in macrophages (*H* = 49.79, *p* < 0.001; Figure 5(a)). Accordingly, TNF-*α* secretion was significantly enhanced after 4 h (*p* = 0.002; Figure 5(a)). *Cyclooxygenase-2* (*Cox-2*) gene expression was already significantly enhanced after 2 h of pressure application (*p* = 0.008) but showed a plateau of upregulation from 4 h (*p* < 0.001) up to 48 h (*p* < 0.001) (*H* = 56.64, *p* < 0.001; Figure 5(b)). In line with that, secretion of COX-2 product prostaglandin E2 (PG-E2) was significantly enhanced as well after 4 h (*p* = 0.002; Figure 5(b)). In strained macrophages, *interleukin-6* (*Il-6*) gene expression was significantly induced already after 2 h (*p* < 0.001) with a maximum effect after 24 h (*p* < 0.001) of compressive force treatment (*H* = 39.77, *p* < 0.001; Figure 5(c)). In analogy, we observed a significantly enhanced IL-6 protein secretion after 4 h of pressure application (*p* = 0.002; Figure 5(c)). Tensile strain in analogy caused a slightly but significantly enhanced gene expression of *Tnf-α* in macrophages after 2 h (*p* = 0.001) and 4 h (*p* = 0.001) of stretching (*H* = 26.20, *p* < 0.001; Figure 5(d)), which could also be reproduced at the protein level after 4 h (*p* = 0.035). In line with that, we also observed an increased gene expression of *Cox-2* already after 2 h (*p* < 0.001) up to 48 h (*p* < 0.001) of tensile strain (*H* = 44.36, *p* < 0.001; Figure 5(e)), with a significant effect on PG-E2 secretion after 4 h (*p* = 0.016). *Il-6* gene expression in and IL-6 secretion by macrophages were significantly enhanced only after 4 h (mRNA: *p* = 0.001; secretion: *p* = 0.004) and 24 h (mRNA: *p* = 0.0013) of stretching (Figure 5(f)).

## 4. Discussion

In our study, we observed effects of tensile and compressive strain on the expression profile of macrophages during simulated orthodontic tooth movement (OTM) indicating a mediating role of these cells during mechanical strain and OTM. Macrophages reacted to compressive or tensile mechanical strain quite quickly with an enhanced expression of proinflammatory cytokines indicating a major role in the sterile immunological response during OTM. In contrast, genes involved in blood vessel formation or extracellular matrix remodelling seem to be mostly regulated by compressive strain.

Compressive force application elicited a time-dependent cytotoxic effect on macrophages, which manifested itself in a reduced cell number and increased LDH release already after 2 h of pressure treatment. As cytotoxic effects may influence the reaction of macrophages to mechanical strain, experiments with pressure application should be reduced to the smallest possible period. A biasing impact of increasing cytotoxicity over time on our expression results cannot be ruled out completely. This increase and associated reduction in cell number, however, were relatively proportional to experimental time, whereas the upregulation of proinflammatory cytokines in particular was quite profound and occurred quite early reaching significance as early as after 2 h in RT-qPCR. This indicates that the observed mechanical strain-induced effects within this short timeframe of 2-4 h are at most only partially affected by the concurrent rise in cytotoxicity. In contrast to macrophages, PDL fibroblasts (PDLF) seem to be more resistant to compressive force treatment, as shown before [10]. In contrast to macrophage cell number being reduced up to 80%, PDLF cell number was only reduced to 50% within 48 h of pressure application [10]. Also, macrophages reacted to pressure application with a 10-fold enhanced release of LDH, whereas PDL fibroblasts only exhibited a 2-fold increase in LDH release after 48 h of compressive force treatment [10] indicating that macrophages react more sensitively to pressure occurring during OTM in the PDL than PDLF. In contrast to our data, studies using human monocyte-derived macrophages reported up to 95% viable cells after 8 h of cyclic pressure application [25]. Tensile strain showed no cytotoxic effects and only a slight reduction in cell number within 48 h in macrophages. In contrast to macrophages, PDLF were reported to show proliferation upon tensile strain due to enhanced transforming growth factor *β* (TGF-*β*) expression [7, 16, 26, 27]. These data indicate that macrophages are more resistant to tensile strain than to compressive strain.

Blood vessels in the PDL are active participants in tissue remodelling associated with OTM, as they are responsible for oxygen and nutrient supply. It is known that due to OTM, there are changes in oxygen supply, as blood vessels are compressed and stretched [7, 27]. VEGF-A is a growth factor involved in the reshaping of blood vessels and angiogenesis [28]. During OTM, the formation of new blood vessels and vasodilatation of existing blood vessels in the PDL are induced at pressure zones [29]. PDLF are known to participate in this reaction, as they enhanced *Vegf-a* expression within 24 h of pressure application [14]. In our study, macrophages also reacted to pressure with enhanced *Vegf-a* gene and VEGF-A protein expression within 2 h to 4 h of treatment, but there were no changes during tensile strain. These data indicate that macrophages participate in and induce neoformation and angiogenesis of blood vessels in compression zones of the PDL during OTM.

Orthodontic forces affect the extracellular matrix of the PDL, as they enhance gene expression of matrix metalloproteinases (MMPs) [7]. MMPs are involved in the breakdown of extracellular matrix in normal physiological processes and tissue remodelling [30, 31]. MMPs are zinc ion-dependent proteolytic enzymes [30], produced by a wide variety of cells during developmental processes [32], inflammatory diseases, degenerative articular diseases [33], tumor invasion [34], and wound healing [31]. MMPs are classified into several subgroups, i.e., collagenases (MMP-1, MMP-8, and MMP-13), gelatinases (MMP-2 and MMP-9), stromelysins, membrane-type MMPs, and other subfamilies. Collagenases like MMP-8 have been shown to be expressed by PDLF [35] and to be upregulated by tensile forces on PDLF occurring within tensile areas of the PDL during OTM [17], whereas they were reported to be downregulated after 24 h, 48 h, and 72 h of compressive force application [17]. We detected a significant upregulation of *Mmp-8* after 24 h of compressive and tensile strain in macrophages. This was in line with previous findings indicating that mechanical factors might participate in MMP expression in macrophages [36]. These data indicate that macrophages in the PDL could also influence MMP expression and thereby affect OTM.

Orthodontic forces induce an aseptic inflammatory response [27]. Production and secretion of proinflammatory cytokines, enzymes, and tissue hormones including TNF-*α*, COX-2, PG-E2, and IL-6 are reported to be involved in tissue reactions associated with OTM [37]. A primary response to orthodontic forces is the synthesis of prostaglandins such as PG-E2 by the inducible cyclooxygenase isoform 2 (COX-2) [11]. Clinical and animal studies have identified a mediating role of COX-2-synthesized prostaglandins for bone resorption [15, 38]. Prostaglandin E_2_ especially seems to be able to mediate inflammatory responses and also to induce bone resorption by activating osteoclastic cells [39]. IL-6 regulates immune responses during inflammation [39] and has an autocrine as well as paracrine effect stimulating osteoclast formation and bone-resorbing activity of preformed osteoclasts [40]. The function of IL-6 overlaps with that of TNF-*α* and IL-1*β*. It was already reported that IL-6 secretion in normal human bone can also be regulated via IL-1*α*/*β* and TNF-*α* [39].

We observed a quite early enhancement of *Tnf-α*, *Cox-2*, and *Il-6* gene expression in macrophages and TNF-*α*, PG-E2, and IL6 secretion by macrophages strained by compressive or tensile forces within 2 to 4 h. PDLF by contrast showed increased gene expression of *Cox-2* and *Il-6* only within 24 h. Our data are in line with previous experiments investigating cyclic pressure application on human monocyte-derived macrophages, which also showed enhanced secretion of TNF-*α* and IL-6 [25]. These data indicate that the synthesis of inflammatory mediators by mechanically stressed macrophages is a very early, initial response to orthodontic compressive and tensile forces, most likely triggering and enhancing the biological mechanisms enabling orthodontic tooth movement.

## 5. Study Limitations and Generalisability

This study gives a first impression of the reaction of macrophages to mechanical stimuli occurring during orthodontic tooth movement. However, there are some limitations associated with this study. We used immortalised murine RAW264.7 macrophages and not bone-marrow-derived macrophages or human peripheral blood mononuclear macrophages. Both cell types could also be used for these experiments but are more difficult in isolation and handling and may show differences according to mouse genetic background or human donor status. Nevertheless, this fact limits generalisability of our results, as transferability to human PBMCs is uncertain. More experiments are needed with macrophages derived from different sources regarding their reaction to mechanical forces. ELISAs were performed with cell culture supernatants only after 4 h of mechanical strain, as no additional information was expected to be gained from also testing protein expression at the other time points. From RT-qPCR results, significant differences to the untreated controls were found as early as after 4 h of mechanical strain with no major expression changes occurring thereafter. The corresponding results from both analyses after 4 h confirm that gene expression of investigated genes is reflected at the protein level—corresponding protein expression levels for the other time points studies in RT-qPCR are thus to be expected.

## 6. Conclusions

Our results indicate that macrophages react early on to tensile and compressive strain occurring during orthodontic tooth movement (OTM) with an enhanced gene expression of proinflammatory cytokines, which could in turn affect PDL fibroblasts, osteoblasts, and immune cells and trigger or enhance the biological mechanisms enabling increased osteoclastogenesis and OTM. Furthermore, macrophages might impact on angiogenesis and remodelling of the extracellular matrix during OTM.

## Figures and Tables

**Table 1:** RT-qPCR gene and primer specifications for reference (*EEF1A1*, *SDHA*) and target genes.

Gene symbol	Gene name	Accession number	5′-forward primer-3′	5′-reverse primer-3′
*Eef1a1*	Eukaryotic translation elongation factor 1 alpha 1	NM_010106.2	AAAACATGATTACAGGCACATCCC	GCCCGTTCTTGGAGATACCAG
*Sdha*	Succinate dehydrogenase complex, subunit A	NM_023281.1	AACACTGGAGGAAGCACACC	AGTAGGAGCGGATAGCAGGAG
*Vegf-a*	Vascular endothelial growth factor a	NM_001287056.1	ACAAGCCTGTAGCCCACGTC	TTGGTTGTCTTTGAGATCCCATGCC
*Mmp-8*	Matrix metalloproteinase 8	NM_008611.4	ACTGATCCTGGTGCCTTGATG	TTGGATGGGGTTGTCTGAAGG
*Mmp-9*	Matrix metalloproteinase 9	NM_013599.4	GTGGGGTTTCTGTCCAGACC	GCACGCTGGAATGATCTAAGC
*Tnf-α*	Tumor necrosis factor-*α*	NM_013693.3	TCGAGTGACAAGCCTGTAGCC	CTTTGAGATCCATGCCGTTGGC
*Cox-2*	Cyclooxygenase-2	NM_011198.4	TCCCTGAAGCCGTACACATC	TCCCCAAAGATAGCATCTGGAC
*Il-6*	Interleukin-6	NM_031168.2	ACAAAGCCAGAGTCCTTCAGAG	GAGCATTGGAAATTGGGGTAGG

## Data Availability

The data used to support the findings of this study are available from the corresponding author upon request.
